# Development and preliminary psychometric properties of a well-being index for medical students

**DOI:** 10.1186/1472-6920-10-8

**Published:** 2010-01-27

**Authors:** Liselotte N Dyrbye, Daniel W Szydlo, Steven M Downing, Jeff A Sloan, Tait D Shanafelt

**Affiliations:** 1 Mayo Clinic College of Medicine, 200 First Street SW, Rochester, MN 55905, USA; 2 Mayo Clinic Department of Health Sciences Research, 200 First Street SW, Rochester, MN 55905, USA; 3 Department of Medical Education (MC 591), University of Illinois-Chicago College of Medicine, College of Medicine, 986 CME, 808 S Wood Street, Chicago IL, 60612, USA

## Abstract

**Background:**

Psychological distress is common among medical students but manifests in a variety of forms. Currently, no brief, practical tool exists to simultaneously evaluate these domains of distress among medical students. The authors describe the development of a subject-reported assessment (Medical Student Well-Being Index, MSWBI) intended to screen for medical student distress across a variety of domains and examine its preliminary psychometric properties.

**Methods:**

Relevant domains of distress were identified, items generated, and a screening instrument formed using a process of literature review, nominal group technique, input from deans and medical students, and correlation analysis from previously administered assessments. Eleven experts judged the clarity, relevance, and representativeness of the items. A Content Validity Index (CVI) was calculated. Interrater agreement was assessed using pair-wise percent agreement adjusted for chance agreement. Data from 2248 medical students who completed the MSWBI along with validated full-length instruments assessing domains of interest was used to calculate reliability and explore internal structure validity.

**Results:**

Burnout (emotional exhaustion and depersonalization), depression, mental quality of life (QOL), physical QOL, stress, and fatigue were domains identified for inclusion in the MSWBI. Six of 7 items received item CVI-relevance and CVI-representativeness of ≥0.82. Overall scale CVI-relevance and CVI-representativeness was 0.94 and 0.91. Overall pair-wise percent agreement between raters was ≥85% for clarity, relevance, and representativeness. Cronbach's alpha was 0.68. Item by item percent pair-wise agreements and Phi were low, suggesting little overlap between items. The majority of MSWBI items had a ≥74% sensitivity and specificity for detecting distress within the intended domain.

**Conclusions:**

The results of this study provide evidence of reliability and content-related validity of the MSWBI. Further research is needed to assess remaining psychometric properties and establish scores for which intervention is warranted.

## Background

Medical school is a profoundly difficult time for physicians in training. Approximately 25% of medical students are depressed [1], 50% experience burnout [2,3], and the majority report quality of life substantially below the age-matched general population [3]. Such distress can have important repercussions for the student and their professional development. Studies demonstrate distress adversely effects competency and professionalism among medical students [4-9] and contributes to illicit drug use [10], marital discord [11], poor physical health/self care [12], and suicide [13,14]. Despite these serious consequences few distressed students seek help [15]. For many students, distress goes unrecognized and untreated and continues into practice [16] where it may have adverse affects on quality of care [17].

These facts highlight the compelling need for medical schools to identify student distress (we use the term *distress *to broadly refer to depression, anxiety, burnout, and related mental health problems). Prompt identification of students in distress would allow schools to identify the challenges faced by their medical student population, provide assistance to those in greatest need, and help prevent the serious consequences by alleviating student suffering. The ability to accurately identify student distress would also allow schools to monitor their learning environment and evaluate the effectiveness of programs intended to promote the well-being of students - accreditation requirements for U.S. medical schools [18].

Unfortunately, existing instruments (e.g. Beck Depression Inventory, Center for Epidemiologic Studies Depression Scale, Maslach Burnout Inventory, Beck Anxiety Inventory, State-Trait Anxiety Inventory) used to assess distress are long, cumbersome to analyze, and typically measure only 1 domain of distress (e.g. depression, burnout, anxiety). In the absence of such an instrument, we have set out to develop a shorter measure of student distress (i.e. the Medical Student Well-Being Index, MSWBI) intended to screen for the common dimensions of distress experienced by medical students and identify the subset of students whose distress places them at risk for serious consequences. This article describes the development of this instrument as well as some evidence for its reliability and validity.

## Methods

The methods consist of a two-stage process. First, in the development stage we identified relevant domains of distress, generated items, and wrote, ordered, and assembled the instrument into a usable form. Second, the judgment stage consisted of expert review of item for clarity, relevance, and representativeness in conjunction with administering the MSWBI to >4000 medical students to obtain data for estimating reliability and validity.

### Stage 1 - Development Stage

#### Identification of domains of distress to be assessed

To complete a thorough literature review to identify common aspects of student distress we repeated the MEDLINE search conducted to inform our systematic review on medical student distress [1] using the time frame of June 2005 to September 2006. Retrieved articles, from both the previous and updated literature review, and relevant articles identified in bibliographies, were read and a list of manifestations of student distress studied was generated. A group of experts (3 authors [LND, JAS, TDS] and 2 external experts [both physicians]) reviewed the list using a nominal group technique [19]. These group members have extensive expertise in the field of medical education, quality of life, instrument development, and the domains of personal (e.g. depression) and professional distress (e.g. burnout). Group members have published extensively in the aforementioned fields. All group members received the list of manifestations of distress identified from the literature review and were asked to comment on these aspects and add any considered to be missing. Responses were collected, summarized, and presented at the consensus meeting where group members decided which aspects should be sent to an Ad Hoc panel of medical school Deans for confirmation of the need for such an instrument and its content.

The Ad Hoc panel of Deans consisted of 8 Deans/Associated Deans from 7 diverse (number of students, geographic location of school, proportion of minority students, private vs. public) medical schools. The Deans were asked: if there is a need for a short instrument to screen for distress among medical students, if the manifestations of distressed selected by the content experts were appropriate, if there were other aspects of distress that should be included, and if any of the domains suggested by the content experts were inappropriate. Additional qualitative feedback was also requested. The nominal group members reviewed the Deans' feedback and finalized the construct definition by consensus.

#### Item generation

Anonymized medical student responses to previously administered comprehensive instruments assessing three of the identified aspects of student distress - burnout (assessed using a slightly modified [word 'work' replaced with 'medical school'] Maslach Burnout Inventory [20]), quality of life (assessed using the Medical Outcomes Study Short Form [SF-8] [21]), and symptoms of depression (assessed using the Primary Care Evaluation of Mental Disorders [PRIME MD] [22]) - were obtained from 2 previous data sets involving >2000 U.S. medical students [2,3]. Multiple studies in the U.S. and abroad have explored the reliability and validity of the Maslach Burnout Inventory [20,23,24], SF-8 [21], and PRIME MD [25].

We calculated Spearman Correlation Coefficients to identify questions on the Maslach Burnout Inventory with the highest correlation coefficient to burnout (defined as having a score of ≥27 on the emotional exhaustion scale or a score of ≥10 on the depersonalization subscale) [20] and emotional exhaustion score and depersonalization score (used as continuous variables). The same procedure was repeated for the SF-8 to identify questions with highest correlation to mental and physical quality of life scores, and for the PRIME MD to identify which question correlated best with screening positive for depression.

For the remaining two aspects of distress- stress and fatigue - a thorough literature review was conduced on MEDLINE for peer-reviewed articles reporting primary data on stress and fatigue in medical students, residents, and physicians. Medical subject heading terms used were combinations of *medical student, resident, intern, internship and residency, physicians*, and *fatigue, sleep deprivation*, or *stress*. The search was limited to articles published in English within the last 10 years (1996 to 2006). Extracted from these articles were the standardized tools used to assess fatigue or stress. A brief review of the non-physician literature on instruments used to assess fatigue, sleep deprivation, or stress was also conducted. The identified instruments were reviewed by nominal group members who selected scales and items that best captured the relevant domain using an iterative process.

#### Instrument Formation

Considering each domain and sub-domain of student distress and the best corresponding item(s) from existing instruments, 7 new items for the MSWBI were written, ordered, and assembled in a usable form by the first author (LND). All response options were a yes/no option consistent with a screening instrument format. These new items were further refined to improve clarity by nominal group members after having evaluated the reasonableness of relationship between the intended domain and new item. As it is important for members of the target audience of the instrument to be involved in content validation of the instrument [26] five 1^st ^through 4^th ^year medical students, who attended medical schools not previously part of our multi-institutional study group, reviewed the new items. These students were asked to comment on the suitability and readability of the items. The students were also asked if other aspects of distress should be included. Some questions were revised to improve clarity based on student feedback.

### Stage 2 - Judgment Stage

The judgment stage consisted of expert review of domain relevance and representativeness of items in conjunction with numerical summarization of the instrument using a statistical index of item-domain congruence [27] and examination for redundancy and internal consistency. Using a standard quantitative approach to evaluate affective measures [26,28,29] 11 experts (7 experts on student psychological distress and 4 experts in undergraduate medical education) from multiple institutions (both U.S. and abroad) independently rated each item on a four-point scale for clarity (i.e., whether there were ambiguities or multiple ways to interpret the question), relevance (i.e. the extent to which each item relates to the aspect of student distress that the item is intended to measure), and representativeness (i.e. how completely the item covers the associated aspect of student distress). Scores for clarity were assessed using basic summary statistics. Following standard approaches [28,29]. Content Validity Index for relevance and representativeness was calculated for each item (the proportion of experts who rate the item as content valid defined as a rating of 3 or 4) and for the entire instrument (computed by averaging the item Content Validity Index across items). Interrater agreement was assessed using overall percent agreement across raters. Pair-wise percent agreement was calculated by averaging the percent of agreement for all possible pairs of raters for each question, adjusted for chance agreement (akin to a kappa coefficient for multiple raters, but more readily interpretable). Assuming a chance agreement rate of 50% we calculated that if observed agreement was outside the 95% confidence interval of 36%-62% then it could be concluded that the amount of agreement would not be due to chance alone. Average pair-wise agreement for clarity, relevance, and representativeness was calculated as an average of the individual item pair-wise agreement percentages. The experts were also asked if any area(s) had been omitted from the instrument. None of the experts involved in this stage were involved in the development of the MSWBI

Lastly, in 2007, 2248 medical students attending Mayo Medical School, University of Washington School of Medicine, University of Chicago Pritzker School of Medicine, University of Minnesota Medical School, University of Alabama School of Medicine, University of California San Diego School of Medicine, and the Uniformed Services University of the Health Sciences completed a web-based survey containing the MSWBI along with the Maslach Burnout Inventory, the PRIME MD, SF-8, the Epworth Sleepiness Scale [30,31], and the Perceived Stress scale [32-34] to measure burnout, symptoms of depression, QOL, fatigue, and stress. Participation was elective and responses were anonymous. Demographic characteristics of participants have been previously reported [14]. From students' responses we calculated Cronbach's alpha, explored for redundancy by calculating the percent of time responders endorsed each possible paired combination [35,36] and the phi coefficient (i.e., a measure of the degree of association between two binary variables that is interpreted similar to correlation coefficient) [37], and estimated the diagnostic accuracy of each item within the intended domain. The latter task was completed by calculating the sensitivity and specificity of each item for detecting distress within the intended domain. Distress within each domain was defined a prior as having high emotional exhaustion (emotional exhaustion score ≥ 27) on the Maslach Burnout Inventory, high depersonalization (depersonalization score ≥ 10) on the Maslach Burnout Inventory, symptoms of depression (positive PRIME MD), low mental or physical quality of life (1/2 standard deviation below the gender and age-matched population norm on the SF-8, a difference considered clinically significant [38]), excessive fatigue (Epworth Sleepiness Scale ≥ 11, a level corresponding to mean scores for patients in need of medical intervention for sleep disorder [31]); or, high stress (Perceived Stress Scale score ≥ 17, a score of half a standard deviation higher than the norm for age-matched U.S. general population) [33].

Institutional review board approval was obtained at each institution involved in the 2004, 2006, and 2007 study prior to surveying of their students. The surveys were administered electronically. On each occasion students were sent an e-mail message with cover letter that informed them about the study and linked to the web-based survey. Participation was elective and all responses were anonymous. The University of Illinois at Chicago institutional review board (this report represents part of LND's thesis work toward a Master's in Health Profession Education from University of Illinois-Chicago) approved the remainder of the study pertaining to instrument development and validation.

## Results

### Stage 1 - Development Stage

#### Identification of domains of distress to be assessed

**Figure 1:**
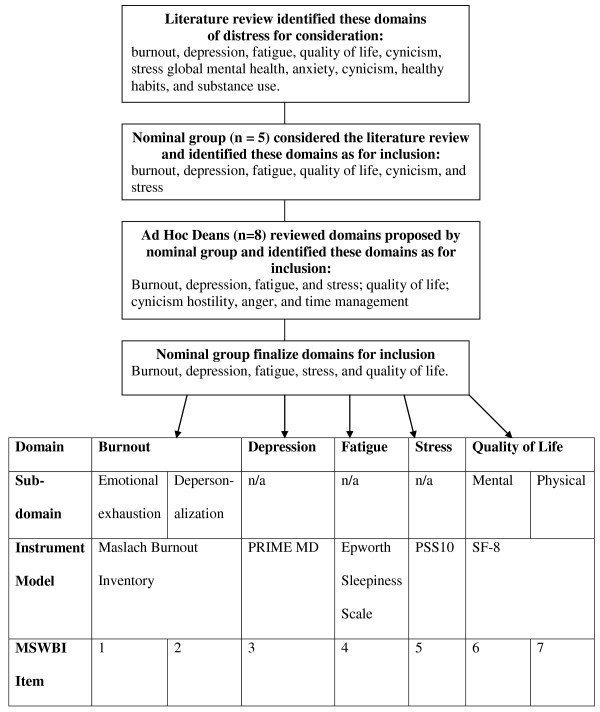
**Process for Domain Identification and Item Generation**. Abbreviations: n/a; not applicable; PRIME MD, Primary Care Evaluation of Mental Disorders; PSS10, Perceived Stress Scale 10-item; SF-8, Medical Outcomes Study Short Form; MSWBI, Medical Student Well-Being Index

From the literature review depression, fatigue, quality of life, burnout, global mental health, anxiety, cynicism, stress, healthy habits, and substance use were identified as aspects of student distress (results illustrated in Figure 1). Nominal group members considered burnout, depression, fatigue, quality of life, cynicism, and stress as the most important aspects of distress to include in the MSWBI. All agreed that substance abuse - though important - should not be included as its inclusion may result in students being less willing to honestly complete the instrument. Seven of 8 Deans (86%) said that there was a need for the screening instrument. Of these 7 all agreed that the burnout, depression, fatigue, and stress should be assessed. Six agreed that quality of life were important and 5 thought cynicism should be included. Additional topics suggested included hostility (1), anger (1), and time management (1). From this information the nominal group members decided common aspects of student distress were burnout, depression, fatigue, stress, and quality of life. Group members endorsed the three dimension construct - emotional exhaustion, depersonalization, and low personal accomplishment - of burnout, as advocated by Maslach [20]; however, as medical professionals are considered to have burnout if they have high emotional exhaustion or high depersonalization [20] the group agreed that emotional exhaustion and depersonalization should be sub-domains considered while the personal accomplishment domain would not be given further consideration. The group decided not to add cynicism separately as it is a dimension of burnout [39].

#### Item generation

The modified-Maslach Burnout Inventory items with the highest correlation coefficient to overall burnout and emotional exhaustion were "I feel burned out from medical school" (r = .66 & .62 and r = .87 & .85, 2004 and 2006 respectively) and "I feel emotionally drained from medical school"(r = .65 & .58 and r = .84 & .82, 2004 and 2006, respectively). The modified-Maslach Burnout Inventory items with the highest correlation coefficient to overall burnout and depersonalization were "I've become more callous toward people since I started medical school"(r = .49 and .51 and r = .83 & .84, 2004 and 2006, respectively) and "I worry that medical school is hardening me emotionally"(r = .53 & .54 and r = .80 & .81, 2004 and 2006, respectively).

For the SF-8 the item "During the past 4 weeks, how much difficulty did you have doing your daily work, both at home and away from home, because of your physical health?" had the highest correlation with physical quality of life (r = .74 & .82,2004 and 2006, respectively) whereas the item "During the past 4 weeks, how much have you been bothered by emotional problems (such as feeling anxious, depressed or irritable)?" had the highest correlation with mental quality of life (r = .94 for both data sets).

Among the PRIME MD questions the item "During the past month have you often been bothered by feeling down, depressed, or hopeless" had the highest correlation coefficients of 0.91 and 0.92.

The nominal group members chose the Epworth Sleepiness Scale among the fatigue instruments identified as it assesses daytime sleepiness and has been previously used in residents and medical students [30,31]. The Epworth Sleepiness Scale item exploring risk of falling asleep while driving resonated with the group as it dovetailed with data on motor vehicle safety in sleep deprived residents [40] and was thought to capture individuals whose degree of daytime fatigue placed them potentially at profound personal risk. After reviewing available tools to assess stress the group identified the question "In the last month, how often have you felt difficulties were piling up so high that you could not overcome them?" on the Perceived Stress Scale [32,33] as most suitable to use as a model for the MSWBI question assessing stress.

#### Instrument Formation

After assembly of the MSWBI consensus was reached among the nominal group members that an obvious and logical relationship existed between the domains and the new items. Group members refined the items to improve clarity. All 5 students reviewed the MSWBI and agreed that 5 of 7 items were suitable for inclusion; one student disagreed with the fatigue item and 2 students thought the item assessing depressive symptoms was redundant with the item assessing global mental quality of life. Three items were identified with poor readability and suggestions for revisions were provided. Based on this feedback, these items were re-written to improve clarity. The students did not identify any additional domains of distress that should be added. The final questions derived from development stage are shown in Table 1.

**Table 1:** Medical Student Well-Being Index*

Item	Question	Domain & Subdomain
1	Do you feel burned out from medical school?	Burnout - Emotional exhaustion

2	Do you worry that medical school is hardening you emotionally?	Burnout - Depersonalization

3	During the past month have you often been bothered by feeling down, depressed, or hopeless?	Depression

4	In the past month, have you fallen asleep while stopped in traffic or driving?	Fatigue

5	During the past month, have you felt that all things you had to do were piling up so high that you could not overcome them	Stress

6	During the past month, have you been bothered by emotional problems (such as feeling anxious, depressed, or irritable)?	Quality of life - Mental

7	During the past month, has your physical health interfered with your ability to do your daily work at home and/or away from home?	Quality of life - physical

* Instrument is copyrighted. Contact author for permission to use.

### Stage 2 - Judgment Stage

The majority of items (no. 2,3,4, and 6, see Table 1) were rated "very clear" or "mostly clear" by all 11 experts. Eight experts (73%) rated item 7 as "very clear" or "mostly clear." Ten experts (91%) rated item 1 as "very clear" or "mostly clear." Item Content Validity Index for relevance and representativeness are shown in Table 2. Item CVI greater than 0.78 is considered excellent regardless of the number of experts [29]. The scale Content Validity Index was 0.94 and 0.91 for relevance and representativeness. Six experts indicated no area had been omitted from the instrument; the remaining 5 indicated one or more of the following should be considered for inclusion: suicidal ideation (2), substance abuse (2), recent life events (1), and social relationships (1). All average pair-wise percent agreement among raters were outside the 95% confidence interval of 35%-62% with the exception of item 7 for clarity (56%) and item 4 for representativeness (49%). Overall pair-wise percent agreement between raters was 89%, 88%, and 85% for clarity, relevance, and representativeness.

**Table 2:** Item level Content Validity Index (CVI) for each of the Medical Student Well-Being Index items*

Item	CVI-relevance	CVI - representativeness
1	1.0	0.91
2	0.82	0.91
3	1.0	1.0
4	0.82	0.64
5	1.0	1.0
6	1.0	1.0
7	0.91	0.91

Overall scale CVI	.94	.91

* Eleven experts (7 experts on student psychological distress and 4 experts in undergraduate medical education) from multiple institutions (both U.S. and abroad) independently rated each item for relevance (i.e. the extent to which each item relates to the aspect of student distress that the item is intended to measure), and representativeness (i.e. how completely the item covers the associated aspect of student distress). CVI for relevance and representativeness was calculated for each item (the proportion of experts who rate the item as representing appropriate content (i.e., content-related validity) defined as a rating of 3 or 4) and for the entire instrument (computed by averaging the item CVI across items).

Using the response data from 2248 medical students the Medical Student Well-Being Index Cronbach's alpha was 0.68. The only scenario in which deletion of one variable increased Cronbach's alpha was when the fatigue item was removed (Cronbach's alpha becomes 0.72). Percent of time responding medical students endorsed each possible paired combination of Medical Student Well-Being Index items (e.g. item by item percent pair-wise agreements) are shown in Table 3. Percent agreements are low and Phi coefficients between all possible pairs ranged from 0.035-0.593 with 14 of 21 combinations less than 0.3, suggesting little overlap of domains and supporting the need to include them all. As shown in Table 4 the majority of MSWBI items had a ≥74% sensitivity and specificity ranged from 63-100% for detecting distress within the intended domain.

**Table 3:** Percent of time responding medical students endorsed each possible paired combination of Medical Student Well-Being Index items

Item	1	2	3	4	5	6	7
**1**	100	9.53	1.89	12.16	8.43	13.73	10.31

**2**		100	2.93	26.02	17.49	28.65	23.19

**3**			100	3.95	2.97	4.28	2.70

**4**				100	26.10	38.81	29.54

**5**					100	28.09	20.84

**6**						100	37.67

**7**							100

**Table 4:** Sensitivity and Specificity of Each Medical Student Well-Being Index Item for Detecting Distress within the Intended Domain

Item	Domain	Sensitivity	Specificity
1: Feel burned out*	Emotional exhaustion	84%	72%

2: Hardened emotionally†	Depersonalization	74%	78%

3: Down, depressed, hopeless‡	Depression	86%	100%

4: Fallen asleep while driving§	Fatigue	11%	99%

5: Things piling up so high¶	Stress	58%	90%

6: Bothered by emotional problems||	Mental quality of life	90%	63%

7: Physical Health**	Physical quality of life	51%	91%

* Sensitivity and specificity of endorsing item no. 1 for high emotional exhaustion (score of ≥27 on the emotional exhaustion subscale score of the Maslach Burnout Inventory). † Sensitivity and specificity of endorsing item no. 2 for high depersonalization (score of ≥10 on the depersonalization subscale of the Maslach Burnout Inventory). ‡ Sensitivity and specificity of endorsing item no. 3 for depressive symptoms (as PRIME MD). §Sensitivity and specificity of endorsing item no. 4 for excessive fatigue (Epworth Sleepiness Scale score ≥11, a level corresponding to mean scores for patients in need of medical intervention for sleep disorder [31]). ¶Sensitivity and specificity of endorsing item no. 5 for high stress (10-item Perceived Stress Scale [33] score of half a standard deviation higher than the norm for age-matched U.S. general population). || Sensitivity and specificity of endorsing item no. 6 (mental quality of life scale of the Medical Outcomes Study Short Form score of ≥1/2 standard deviation below the gender and age-matched norm, a difference considered clinically significant [38]). **Sensitivity and specificity of endorsing item no. 7 (physical quality of life scale of the Medical Outcomes Study Short Form score of ≥1/2 standard deviation below the gender and age-matched norm, a difference considered clinically significant [38]).

## Discussion

Distress is pervasive among medical students with some students suffering serious consequences. Given the high prevalence of distress it may be impractical or impossible to intervene on an individual level with every student in distress. Ideally, schools need to be able to identify students with degrees of distress that may place them at risk for serious personal or professional consequences. Given the impracticality of administering existing long diagnostic instruments evaluating the individual domains of distress and the reluctance on behalf of students to seek help for their distress a short, pragmatic instrument that screens for the common dimensions of distress experienced by medical students and identifies students at greatest risk for serious personal and professional consequences would be of use to medical schools. Using appropriate methods of content development [26,28] we developed such an instrument, intended to screen for common dimensions of distress experienced by medical students and in this report describe steps taken to ensure its validity.

Although the MSWBI needs further validation before widespread use can be recommended, it has the potential to be a useful tool for medical schools and their students. Unlike long surveys designed to be diagnostic tools to evaluate individual domains of distress, the MSWBI has only 7 items, covers multiple aspects of distress, and is easy to administer and complete. For screening questionnaires to be helpful they need to be brief, easy to administer, valid, reliable, and sensitive. Content-related evidence, response process validity evidence, and internal structure validity evidence are part of a broader set of construct-related evidence [27]. Considerations of content are extremely important during instrument development as the domain and extensiveness of domain coverage influence and limit the specifics of score inferences supported by other evidence [27]. This study provides strong content-related validity evidence for the 7-item MSWBI. Our rigorous development strategy employed a thorough literature review and involvement of a nominal group of experts, an Ad Hoc group of Deans, and medical students during conceptualization of the constructs included (i.e. important aspects of distress). This makes substantial construct under representation and construct irrelevance less likely. Item writers were well qualified and 11 international, independent experts considered the MSWBI items clear, relevant, and representative with solid inter-rater agreement. The sensitivity and specificity of most of the MSWBI provides further evidence of an empirical relationship between the items and the intended domain. Based on the evidence presented it is reasonable to conclude that the content-related validity of the MSWBI is satisfactory when used as a brief screening instrument [26]. Response process validity evidence, although not directly ascertained, is likely to be adequate as students are familiar with the survey format and as a professional survey research center administered the web-based survey and electronically transferred data into a format analyzable by our statistical software, thereby minimizing data entry error and ensuring quality control. Internal structure validity is suggested by the low percent of time responding medical students endorsed each possible paired combination of MSWBI (i.e. low percent pair-wise agreement) and the low Phi, both proxies of inter-item correlations. The MSWBI has moderate reliability that improves after removal of the fatigue item, a finding of possible relevance as our experts did not consider the fatigue item as well representative of its domain in comparison to other items. As routine driving may be limited to medical students in North America this item may not be well suited for a distress screening index. Nonetheless, the fatigue item should be retained until further studies are carried out.

This study has several limitations. First, distress is a multi-dimensional construct. We may have left out some aspects of distress that are important, or included some that should have been left out, or both [27]. Second, we did not have data to allow for identification of single items that best correlated with high daytime fatigue scores or high perceived stress among medical students. Rather we relied on a thorough literature review and an iterative process to identify items most likely to capture the intended aspect of student distress. Third, the item and scale CVI does not adjust for chance agreement [29]. The CVI, however, has advantages over other calculations of inter-rater agreement as they capture agreement of any type (including agreement about low relevance of an item). Although the probability of chance agreement is low as this study involved 11 experts [29] we also calculated average pair-wise agreement between raters with resulting data supporting that agreement was not due to chance alone. Finally, the fatigue item was not considered well representative of its domain by our experts, and removal of the fatigue item improves the internal consistency of the MSWBI. Nonetheless, we elected to retain the fatigue item, until further validity studies are carried out to determine its ability to identify clinically relevant fatigue among medical students.

There are also several strengths of our study. First, to our knowledge, this is the first study to use established and validated methods to develop a screening instrument for distress among medical students. Second, we used a rigorous and standard process for development and judgment of the instrument [26,28]. We carefully defined the domains for inclusion with input from experts, deans, and medical students, thoughtfully generated items, involved multiple judges from diverse medical schools in the U.S. and Europe, quantified judgments using formalized scaling, and explored for redundancy of items and internal reliability [26]. We calculated item and scale CVI which allows for focus on consensus rather than consistency estimates and used an adequate number of content experts during the judgment stage [28]. The CVI has several additional advantages such as being easy to compute, understand, and communicate and providing item diagnostic information and scale validity information [29]. The approach we took to quantify judgment of the instrument using a statistical index of item-domain congruence is advocated by Messick [27] and commonly used to evaluate affective measures [26,28,29,41]. Third, average pair-wise percent agreement obtained among expert raters suggest a low probability of agreement due to chance alone, lending support for interrater agreement. Fourth, from a practical perspective we reduced concepts previously assessed in five different instruments with a total of 50 questions to a 7-item screening index.

## Conclusion

To our knowledge, this is the first study to use established and validated methods to develop a screening instrument for distress among medical students. Given the importance of well-being in physicians across training this brief 7-item instrument has potential to be useful. The ability of the MSWBI to identify those individuals whose distress is placing them at risk for serious adverse consequences such as suicidal ideation, leaving medical school, or substance abuse warrants further exploration. Early identification of students whose degree of distress places them at risk for adverse consequences is vital to ensuring prompt intervention and appropriate allocation of resources to assists those in greatest need. The MSWBI is a promising screening tool to identify medical student whose degree of distress places them at risk for adverse outcome. Continued research is needed to further validate and refine the MSWBI to maximize its sensitivity and usefulness as a screening instrument.

## Abbreviations

(CVI): Content Validity Index; (ESS): Epworth Sleepiness Scale; (MSWBI): Medical Student Well-Being Index; (SF-8): Medical Outcomes Study Short Form; (PSS10): Perceived Stress Scale 10-item; (PRIME MD): Primary Care Evaluation of Mental Disorders; (QOL): Quality of life

## Competing interests

The authors declare that they have no competing interests.

## Authors' contributions

LND conceived of the study, acquired the data, interpreted the data, drafted the manuscript and revised it. DWS and JAS acquired the data, conducted the data analysis, interpreted the data, and revised the manuscript. SMD participated in study design and interpretation of data and revised the manuscript. TDS conceived of the study with LND, interpreted the data, and critically revised the manuscript. All authors approved the final manuscript.

## Authors' information

**Liselotte N. Dyrbye, MD MHPE **is assistant professor of medicine, Mayo Clinic College of Medicine, Department of Medicine. Dr. Dyrbye is the Associate Director, Research Applications, Mayo Clinic's Program on Physician Well-being. She has 24 peer-reviewed publications of which 11 pertain directly to trainee or physician well-being.

**Daniel W. Szydlo, BA **was a statistician, Mayo Clinic Department of Health Sciences Research at the time of the study. He is now a graduate student at University of Washington, Seattle, Washington.

**Steven M. Downing, PhD **is associate professor of medical education, University of Illinois-Chicago College of Medicine, College of Medicine. Dr. Downing's research interest in educational measurement and assessment in medical education has resulted in more than 100 research papers, book chapters, and presentations.

**Jeff A. Sloan, PhD **is professor of oncology, Mayo Clinic Department of Health Sciences Research. Dr. Sloan is statistician with extensive expertise in quality of life research.

**Tait D. Shanafelt, MD **is associate professor of medicine, Mayo Clinic College of Medicine, Department of Medicine. Dr. Shanafelt is the Director of the Mayo Clinic's Program on Physician Well-being and has published extensively in the field of trainee and physician well-being.

## Pre-publication history

The pre-publication history for this paper can be accessed here:

http://www.biomedcentral.com/1472-6920/10/8/prepub
